# Mobile genomics: tools and techniques for tackling transposons

**DOI:** 10.1098/rstb.2019.0345

**Published:** 2020-02-10

**Authors:** Kathryn O'Neill, David Brocks, Molly Gale Hammell

**Affiliations:** 1 Simons Center for Quantitative Biology, Cold Spring Harbor Laboratory, Cold Spring Harbor, NY 11724, USA; 2 Department of Computer Science and Applied Mathematics, The Weizmann Institute of Science, Rehovot, Israel

**Keywords:** transposable elements, computational genomics, retrotransposons, single-cell analysis

## Abstract

Next-generation sequencing approaches have fundamentally changed the types of questions that can be asked about gene function and regulation. With the goal of approaching truly genome-wide quantifications of all the interaction partners and downstream effects of particular genes, these quantitative assays have allowed for an unprecedented level of detail in exploring biological interactions. However, many challenges remain in our ability to accurately describe and quantify the interactions that take place in those hard to reach and extremely repetitive regions of our genome comprised mostly of transposable elements (TEs). Tools dedicated to TE-derived sequences have lagged behind, making the inclusion of these sequences in genome-wide analyses difficult. Recent improvements, both computational and experimental, allow for the better inclusion of TE sequences in genomic assays and a renewed appreciation for the importance of TE biology. This review will discuss the recent improvements that have been made in the computational analysis of TE-derived sequences as well as the areas where such analysis still proves difficult.

This article is part of a discussion meeting issue ‘Crossroads between transposons and gene regulation’.

## 1. Introduction

While several types of genomic repeated sequences exist, the largest fraction of the human genome, approximately half, is comprised of transposable elements (TEs) [1], though some groups estimate much larger TE fractions [2]. These TEs, often called transposons or jumping genes, are DNA sequences that have, or once had, the ability to mobilize within the genome, either directly or through an RNA intermediate. TEs are present, to varying degrees, in the genomes of all known types of organisms, both prokaryotic and eukaryotic, with some species showing more genomic transposons than host sequences [3]. Several excellent reviews have discussed the many and varied types of TEs [4–6]. Briefly, TEs come in two major types. Class I TEs, also called retrotransposons, first transcribe an RNA copy that is then reverse transcribed to cDNA before inserting elsewhere in the genome. Class II TEs, also called DNA transposons, directly excise themselves from one location before reinsertion. In the human genome, the vast majority of TEs are of Class I, retrotransposon type. Nearly, all human TEs have lost the ability to fully mobilize [7–9], with the human-specific LINE-1 element (L1HS) being the only fully autonomous TE with the ability to generate new transposition events to date. However, most TEs have retained some level of functionality, including the ability to direct their own transcription. Thus, transcriptome-wide sequencing assays, like RNA-seq, frequently include transposon-derived transcripts among the set of expressed sequences. Moreover, some transposon transcripts have been co-opted to play a role in host function, particularly during early development, such that some expressed transposon transcripts have been shown to be necessary for proper cell differentiation and maintenance of identity [10–14]. In addition to their roles in general cellular function, several types of transposons have become intricately entangled within gene regulatory networks [15], contributing both to *cis*-regulatory sequences [16–18] as well as general chromatin environments [19–21]. For this reason, it is paramount, we consider the contribution of repetitive elements as we unravel the genomic and epigenomic landscapes that control gene expression.

Properly accounting for repetitive regions in most genomics analysis settings requires special considerations for the challenges presented by the number of nearly identical transposon sequences dispersed throughout our genomes. Thus, reads derived from these regions are frequently discarded in most sequencing data analysis protocols owing to the difficulty in properly assigning TE-derived reads to the correct locus of origin. Few packages explicitly support inclusion of repeats and some intentionally discard reads from these regions, as discussed in a recent review [22]. Of the packages designed to address TEs, many tools focus on the detection of novel TE insertions or TE-associated genomic rearrangements. Few tools are developed specifically to address regulatory and transcriptional activity of TEs in common assays, such as RNA-seq, chromatin immunoprecipitation sequencing (ChIP-seq), cross-linking immunoprecipitation sequencing (CLIP-seq) and small RNA-seq (sRNA-seq). In this review, we seek to provide an overview of the packages that explicitly support the inclusion of TE sequences in differential expression and binding analyses, and the strides which have been made to improve our ability to resolve ambiguously mapped reads in genomics analysis.

## 2. Annotation and de novo detection

A well-assembled and annotated genome is the foundation for effective analysis, as all subsequent analyses discussed below require a reference genome as well as a map of gene and TE positions. While many genomes have near-complete assemblies, and extensive annotation, the quality of both tends to drop over repeat-rich regions for the same reasons discussed above: ambiguity in placing near-identical sequence reads from highly similar copies of related transposons. This ambiguity leads to non-contiguous and erroneous chromosomal assembly, which will feed forward into any genomics analyses using these assemblies [23]. Genome assembly has benefitted immensely from long-read sequencing technologies, particularly in the context of highly repetitive centromeric regions and in nested repeating elements [24,25]. While these long-read technologies are improving the reference genomes used to map new datasets, one caveat is that transposons are often polymorphic within populations, such that each new sample sequenced is expected to have many non-reference transposon-associated insertions, deletions and other structural variants that may be rare or private [26,27].

Once a high-quality assembly is constructed, the process of annotation may begin. Many curated annotation databases have been developed for identifying repeat elements. For an in-depth review of annotation practices and existing repositories, please refer to the review by Goerner-Potvin *et al*. [22]. Here, the distinction between TE-, genome- and polymorphism-focused annotation repositories is emphasized in addition to a list of software for de novo insertion detection. The most widely used database of TE consensus sequences is RepBase [28], which provides the sequences with which genome-specific annotation files are constructed. These annotation files are available through the University of California Santa Cruz Genome Browser (UCSC) and RepeatMasker [29]. While new RepBase consensus sequences require a subscription, several open databases for repeat annotation are available in addition to UCSC including: RepetDB [30], ERVdb [31], Dfam [32], TREP [33], SPTEdb [34], ConTEdb [35] and mips-REdat [36]. The ideal database for analysis will vary depending on the model organism and TEs of interest, as some databases are species and TE type-specific.

## 3. Mapping

**Figure 1.**
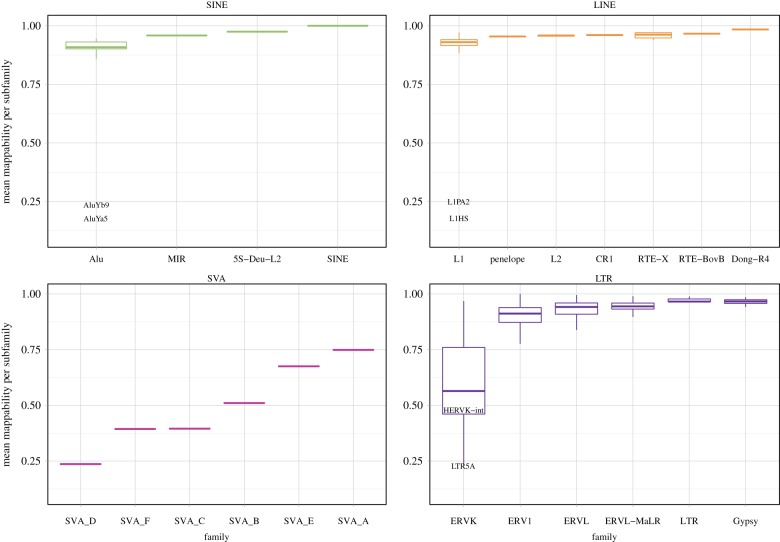
Estimated mean mappability for different types of TEs in the human genome. Mappability tracks from the analysis by Sexton and Han for hg38 were used to construct mean mappability estimates (average probability that a pair of 76 bp reads would map uniquely to a genomic instance of that TE). These were then aggregated by subfamily (L1HS is a human-specific subfamily of the LINE class). Some TEs have accumulated enough mutations across each locus that nearly all copies are uniquely mappable. Very recently inserted, and/or still active TEs, show the lowest mappability rates with many copies still very close to the consensus sequence (e.g. Alu and SVA types). By contrast, many older SINE and LINE TEs have high mappability rates and can easily be assessed using only uniquely aligning reads with standard analysis procedures. Mappability was calculated by counting number of times a 76 bp paired end read (242-mer with an internal gap of 100 nt) would map within the genome at a particular nucleotide where that nucleotide was the beginning of a 242-mer.

After the construction of a well-annotated reference genome, one is faced with the task of mapping experimental data to the appropriate reference. Even with a perfectly annotated and constructed genome, ambiguously mapped sequencing reads still present a challenging problem. One of the first approaches to address this problem, designed for RNA-seq analysis, was to probabilistically assign multi-mapped reads to regions that also show a higher density of uniquely mapped reads, i.e. reads with a single best genomic alignment under the mapping software's heuristics [37]. However, this was a highly gene-centric model that was primarily focused on host gene expression, and was not explicitly intended for estimating expression from TE loci. Moreover, this approach is biased towards regions that have some uniquely mappable content. Unfortunately, the most recently integrated TE insertions are also the least likely to be uniquely mappable, and are thus the most likely to be lost or underestimated by these methods. To highlight this, figure 1 displays the estimated mappability of several different types of TEs in the human genome, with a specific emphasis on younger types of TEs shown to be active in the human genome [38]. Mappability in this plot was defined as the inverse of the number of times a simulated 76 bp paired-end read mapped to the genome, allowing three mismatches. Mappability was scored per nucleotide with the score assigned to the first nucleotide of the read. This track was procured from an in-depth analysis performed by Sexton and Han which considers the many parameters that contribute to the mappability of a particular sequence, including the mapping software chosen and the length of the sequenced read [39]. These analyses still return to the same basic theme displayed in figure 1: mappability rates vary for different types of transposons, and the most recently inserted transposons are the most likely to be discarded by standard analyses that rely on uniquely mapped reads. In other words, the transposons that present the most problems in genomics analyses are precisely those that are more likely to be functional in terms of: carrying fully functional promoters, encoding for functional proteins, and, rarely, mobilizing within the genome. In addition, many older elements with degraded versions of these components have been recycled to play roles in *cis*-regulatory architecture [40].

Most genome alignment software is aware of the difficulties posed by ambiguously mapped reads, and thus provide extensive parameter sets designed to allow the user to choose the number of alignments considered for each sequenced read. This includes standard genome mapping software applicable to genome resequencing studies as well as ChIP-seq-based studies of protein-DNA binding, such as BWA [41], Bowtie [42] and Novoalign (http://novocraft.com/). For RNA-seq aligners, there are two approaches, those that align to reference transcriptomes and those that align to genomes. Transcriptome methods like kallisto [43] and Salmon [44] perform pseudoalignments with transcript derived k-mers and can attempt to build the reference transcriptome from the RNA-seq data itself. Salmon can be specified to report unmapped reads, kallisto does not include this option. While pseudoalignment is very fast, computationally less intensive, and helpful in organisms without a reference genome, it can be complicated in the context of repetitive elements, where all of the caveats that make genome assembly difficult (discussed above) would also apply to de novo transcriptome assembly. With regard to genome-based RNA-seq aligners, there are a number of packages available including: STAR [45], HISAT2 [46], GSNAP [47], Novoalign, RUM [48], Minimap2 [49] and others [50]. In the context of sRNA-seq data, short-read genome-based aligners (BWA [41], Bowtie [42] and SCRAM [51]) that do not consider splice junctions tend to work as well or better than RNA-seq tailored algorithms, with SCRAM being specifically designed for small RNA analysis pipelines. Another approach to improve mappability would be to incorporate long-read sequencing methods, as longer reads contain more information and can serve as a way to reduce ambiguity in the context of RNA-seq. Many of the previous aligners like STAR, HISAT2 and GSNAP have been applied to long-read sequencing data after error correction [52] and have been shown to work well. In addition, algorithms like BLASR [53], GraphMap [54], rHAT [55], LAMSA [56], Kart [57], NGLMR [58] and lordFAST [59] have been developed specifically to address the increased length and error rates associated with long-read technologies.

Some tools designed to improve mapping rates for repetitive regions work after an initial analysis with one of the tools listed above. These standalone tools can use alignment files as input and then attempt to statistically redistribute the ambiguous reads based on distributions of neighbouring alignments. One such algorithm is MMR [60] which iteratively redistributes ambiguously mapped reads across their respective loci to maximize smoothness of multimapped read distribution in the context of unique reads, or reduce the variance in coverage. Another is a Gibbs sampling method [61] which uses stochastic redistribution of multimapped reads, normalized to the background distribution, in order to iteratively search for the most likely locus of origin. This type of iterative statistical technique for optimal assignment of reads to the correct loci has been picked up and elaborated on by several different groups, and represents a theme throughout the review. While it does not employ the statistical redistribution of reads, CoCo [62] is a package which corrects and salvages multimapped reads by taking into consideration nested genomic architecture, a common feature associated with TEs.

## 4. Analysis

**Figure 2.**
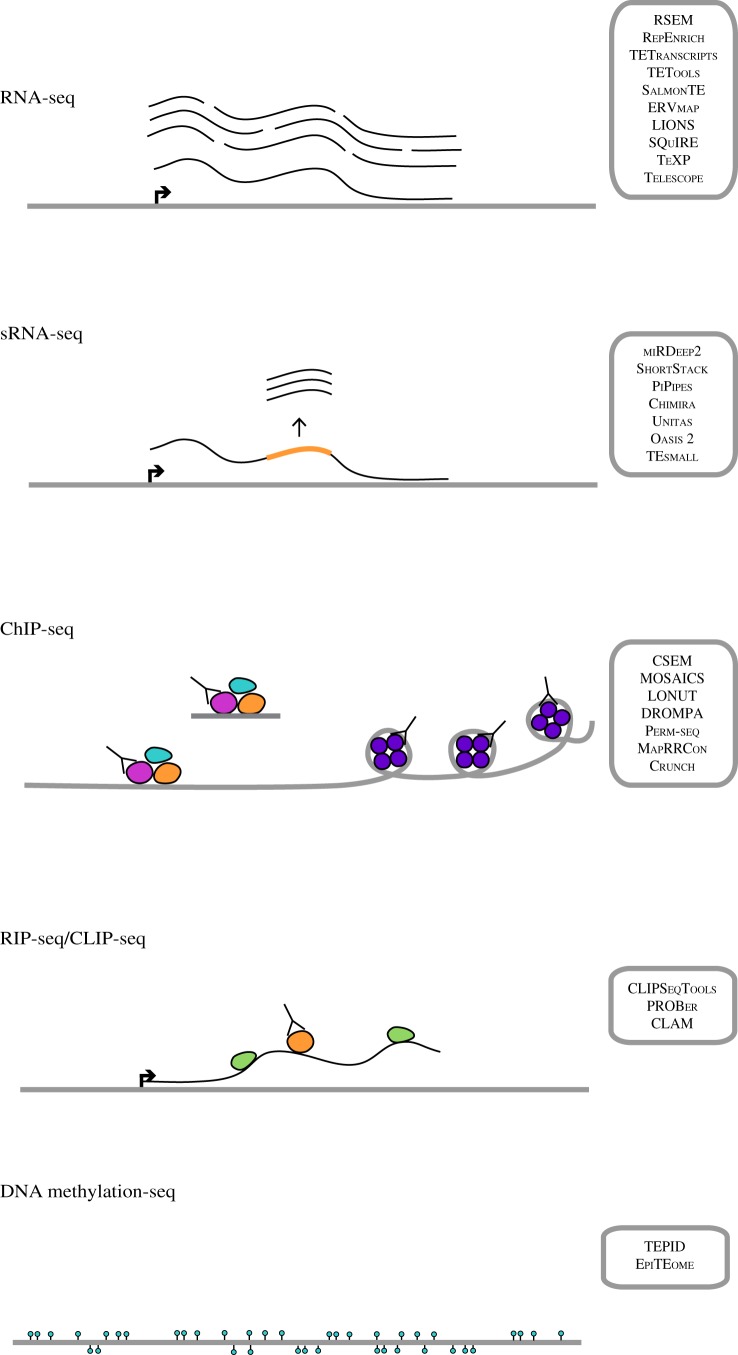
Published tools available for including repetitive regions in several common genomics analysis protocols. These have been divided into those that are geared towards RNA expression analysis (RNA-seq), small RNA expression analysis (sRNA-seq), genome and chromatin binding factors (ChIP-seq), RNA-binding factors (RIP/CLIP-seq) and DNA methylation analysis (DNA methylation-seq) A table describing these tools (electronic supplementary material, table S1) also provides links and references for the software and associated publications.

The next step in a general next-generation sequencing (NGS) sequencing analysis pipeline is to annotate and quantify those reads which mapped to the genome. The mapping profiles will vary widely based on molecular context of the sequencing library. Each type of NGS data comes with its own challenges in the context of highly repetitive elements. The remaining sections will go through analysis strategies for each of the most common NGS data types in detail. The tools in these sections are listed for reference in figure 2, where they are grouped by the experimental assays used to generate the data. Electronic supplementary material, table S1 gives references and links to the software for all tools described.

## 5. RNA-seq

RNA-seq for expression analysis is one of the most well-studied areas in genomics, and this is also reflected in the diversity of tools available for analysis of transcripts from repetitive regions. RNA-seq data derived from short-read sequencing platforms is comprised of small fragments, derived from short single- or paired-end reads tiled across the region of a transcript of origin. Of the tools which have been developed to facilitate transcriptional analysis of repetitive elements, here we will focus on those which take into consideration ambiguously mapped reads. How to address ambiguously mapped reads is an old problem in genome science particularly when using older sequencing technologies from which reads were much shorter (approx. 36 nt) than what we currently consider a short read (approx. 150 nt). These early RNA-seq packages were largely gene-centric, as investigation of repetitive elements with these earlier technologies was (and remains) a challenge. However, the basic principles for probabilistic redistribution of ambiguously mapped reads emerged at this time. The first strategies employed a single-step multimapped read redistribution based on the number of uniquely mapped reads at each locus. [37] This was followed quickly by an expectation-maximization (EM) algorithm to iteratively estimate the most likely expression levels of gene transcripts based on relative counts of unique and multimapped reads [63]. In addition to probabilistic redistribution of reads, packages like Cufflinks [64] and HTseq [65] have multimapper modes where ambiguously mapped reads are weighted by the relative number of genomic alignments (as 1/*n*, where *n* is the number of potential alignments in the genome). The package Scavenger [66] considers multimapped reads and uses an intermediate consensus assignment with remapping to rescue unmapped reads. Differences in strategies used to address multimapped reads and their associated limitations are outlined in detail by Treangen & Salzberg [23].

As interest broadened to begin investigating transposon expression through RNA-seq explicitly, several packages were developed to handle transposons separately from the rest of the transcriptome. Among the first TE-centric packages was RepEnrich [67] which functions by creating repetitive element pseudochromosomes, which are a series of contigs that represent all of the genomic instances of each transposon subfamily annotated in RepeatMasker, concatenated onto a single region. These subfamily pseudochromosomes were then used to identify reads that mapped only to one subfamily of transposons, such as the human-specific LINE element L1Hs, even if the exact generating locus was still ambiguous. This was able to separate the level of uncertainty to finer detail, such that reads could be described as: unique in the genome, unique to a particular subfamily or ambiguously mapping to multiple types of transposons. Similar to RepEnrich, TETools [68] is another transcript quantification method which uses a detailed annotation file or ‘rosette’ to facilitate quantification from TE-derived reads, and which again aggregates reads at the subfamily level. TeXP [69] is a package which focuses on LINE-1 elements specifically and models spurious genome transcription to more accurately quantify LINE-1 expression. TEtranscripts [70] was the first TE-centric algorithm to implement statistical read redistribution to handle multimapped reads. TEtranscripts uses an expectation maximization algorithm to find the most likely distribution of ambiguously mapped TE-derived RNA-seq reads, and also includes expression estimates for both host genes and TEs in the output. After TEtranscripts, other packages have been developed to expand the methods used for statistical read redistribution including MMR [60] and SalmonTE, [71] with SalmonTE being unique in its use of a pseudoalignment strategy from the authors of the original Salmon [44] package in order to bypass the mapping step typically used in RNA-seq analysis. Yanagi [72] expands on this pseudoalignment strategy by mapping to a segmented version of the transcriptome to reduce ambiguity of mapping.

In the packages described above, quantification was performed at the subfamily level, as determining the specific expressed genomic loci within a subfamily is quite difficult for TEs that are close to the consensus sequence. However, several newer packages have been released to address the need for locus-specific quantification of TE-derived transcripts. TE-centric packages include SINEsFIND [73], and ERVmap [74] which are specialized for their respective TE family of interest. Two pipelines used genome-guided de novo transcriptome assembly with Trinity [75] to quantify TE expression at a locus-specific level: TEcandidates [76] and a pipeline described by Guffanti *et al*. [77] More recently, SQuIRE [78] (software for quantifying interspersed repeat expansion), and Telescope [79] adapted the EM-based read redistribution strategies described above to infer originating loci of ambiguously mapped reads, using uniquely mapped reads surrounding the locus to guide the EM read redistribution.

One of the motivating reasons to study TEs is for their influence over regulatory networks in our genome. To address this specifically, a final type of RNA-seq analysis package has been released at the interface of gene-centric and TE-centric models. LIONS [80] is a novel package which detects novel fusion events that connect TE promoter sequences to downstream coding gene sequences. These chimeric TE/gene transcripts represent one of the many ways that TE promoter elements might affect regulation of adjacent genes.

## 6. Small RNA-seq

Cells regulate TE expression using multiple strategies. The most potent silencers of TEs in germline cells are small RNAs (sRNAs) of the PIWI-interacting RNA (piRNA) class [81]. In somatic tissues, two additional classes of small RNAs contribute to TE silencing: short interfering RNAs (siRNAs) derived from expressed transposon transcripts [81] and the more recently described 3′ tRNA derived fragments (3′ tRFs) [82]. Therefore, it is integral to the study of transposon biology to consider sRNAs and accurately quantify their production. To this end, several packages have been released to investigate sRNA species, which prove particularly challenging when derived from repetitive loci in the genome as they are short in length, typically between 18–36 nucleotides. Packages like MiRdeep2 [83], ShortStack [84], PiPipes [85], Chimira [86], sRNAtoolbox [87], Oasis 2 [88] and Manatee [89] have been developed to detect specific types of sRNA loci in the genome and quantify their differential expression. While microRNAs (miRNAs) are not known to play a large role in transposon regulation, a large fraction of miRNAs and other known TE regulatory sRNAs are present in multiple copies in the genome, making TE-focused strategies for multimapped read resolution useful, even for non-TE-derived sRNAs. Statistical techniques, including machine learning, have already been extensively employed in the arena of piRNA prediction, a critical step for the ultimate quantification of piRNA reads accumulation in packages like piRNAPredictor [90], Piano [91] and a k-mer-based method described by Zhang *et al*. [92] ShortStack after publication was updated to include Butter [93] which now performs statistical redistribution of multimapped reads.

These methods described above have largely considered sRNA classes separately, however, several packages including Unitas [94] and TEsmall [95] have strived to consider sRNA classes comprehensively to facilitate proper normalization of heterogeneous sRNA libraries, and to facilitate differential expression analysis across classes while taking into consideration ambiguously mapped reads.

While several iterative statistical methods have been employed in the study of sRNAs for annotation and target prediction [96], there is still much room for improvement in the handling of ambiguously mapped reads for small RNA expression analysis. Many of these issues have been nicely reviewed by Bousios *et al*. [97] particularly in the context of plants whose genomes are highly enriched in TEs and where sRNAs form a large component of the TE silencing machinery. Briefly, the chief challenge for applying probabilistic read redistribution algorithms for sRNA loci is that many types of sRNAs accumulate as very short transcripts cut from larger precursors. Often the precursors are rapidly processed and/or would not be caught by sRNA library preparation protocols. For miRNAs, for example, typically only the guide and passenger strands are detected in sRNA-seq libraries, leaving only two short approximately 22 nucleotide sRNAs and few surrounding reads from the precursor transcript to help guide decisions about the true originating locus. Thus, some loci may be more amenable to statistical inference algorithms, while others need additional assays in order to determine the precise source of sRNA biogenesis.

## 7. Immunoprecipitation-sequencing (ChIP, CLIP and RIP)

In this section, we have grouped together multiple disparate genomics data types that all involve immunoprecipitation-based steps in order to find protein binding sites in nucleic acids. These data can be derived from chromatin-bound factors (ChIP-seq) or RNA-binding proteins (CLIP-seq/RIP-seq), but are grouped here as IP-seq because of the similar challenges these data types present for computational analysis pipelines. Typically, the published pipelines for IP-seq data analysis begin by discarding multimapped reads in order to achieve higher specificity and resolution for the protein binding sites. This can be troublesome when studying proteins which bind to regions rich in repetitive elements. For example, H3K9me3 histone markers are known to be enriched in constitutive heterochromatin [98], a region of the genome highly enriched in repeat elements. Therefore, when calling H3K9me3 peaks using only uniquely mapped reads, the actual enrichment above background levels may be significantly higher than what is reported, skewing the estimates of background levels and discarding many truly bound regions. While this is a known issue for heterochromatin binding proteins, recent surveys of DNA- and RNA-factors have shown that transposon-derived regulatory elements form a significant fraction of both transcription factor binding sites [18,99] as well as RNA-binding protein recognition elements [100,101].

For ChIP-seq-based datasets, it is important to acknowledge the differences and difficulties associated with attempting to detect binding elements for chromatin binding factors and marked histones that typically bind broadly over large areas (broad peaks) when compared with transcription factors, which typically display sharp, narrow peaks. H3K9me3 typically shows a broad peak profile, as these histone marks are found on nucleosomes spread across wide stretches of chromatin. This distribution warrants a different detection strategy than that used for a typical transcription factor, such as MYC, which might occupy narrow binding regions, on the order of approximately 50–150 nucleotides in a typical assay. This is particularly relevant when these different peaks occur in repetitive genomic regions. The larger the bound region, the more likely it is that some of that genomic sequence will be uniquely mappable, which can guide the inference about read accumulation in adjacent sequences.

To address multimapped reads specifically, packages like the peak caller CSEM [102] have used expectation maximization to redistribute ambiguously mapped ChIP-seq reads based on the distribution of surrounding uniquely mapped reads. Owing to the reliance on uniquely mappable reads, these methods function best on broader peaks because they query a larger region, which may be more likely to contain uniquely mappable content. LONUT [103] calls a set of unique peaks and a set of non-unique peaks, then aggregates both call sets together to remove any redundancy. MOSAiCS [104], while not specifically developed to handle repetitive regions, recommends using the CSEM algorithm as a pre-processing step in order to include multimapped reads. DROMPA [105] and Crunch [106] take into account multimapped reads using a simple 1/*n* fractional distribution strategy. Crunch subsequently places a large emphasis on motif prediction and annotation. The analysis pipeline MapRRcon [107] uses unique and multimapped reads, but resolves the issue of multimapped read ambiguity by calling peaks on the consensus sequence of transposon subfamilies.

There is still significant room for progress in the arena of ChIP-seq analysis in repetitive regions. It is still difficult to call narrow peaks in repetitive regions, owing to the lack of sufficient reads surrounding the locus of interest to guide the inference algorithms. Perm-seq [108] addresses this issue by using the orthogonal dataset of DNAase hypersensitivity profiling for better resolution in repetitive regions of the genome. As sufficient reference datasets become available in multiple cell types and conditions, this may make this strategy feasible as a general method. By contrast, while broad peak callers tend to include more information within the locus of interest to help guide inference across repetitive regions, the data from these methods tend to have a lower signal-to-noise ratio, such that improvement of broad peak callers generally is still an active area of computational development.

The problems described above in the context of ChIP-seq analysis are compounded in the context of CLIP- and RIP-seq datasets, where one must also normalize for differences in the expression level of the bound transcript substrates. If the bound transcripts contain repetitive regions, or are entirely composed of repetitive elements, one must first find a way to accurately distribute ambiguous reads among the input transcriptome dataset before calling enriched binding sites in particular transcripts. CLIPper [109] was one of the first CLIP-seq pipelines, but was restricted to uniquely mapped reads only. CLIPSeqTools [110] is a CLIP-analysis pipeline which randomly assigns ambiguously mapped reads to one of their candidate mapping loci. CLAM [111] uses expectation maximization algorithms, as described above, to redistribute ambiguously mapped reads between expressed transcripts, but the algorithm works only on the alignment file and does not include information about enriched peaks in its statistical weights. PROBer [112] has been developed as a general-purpose algorithm for detecting sites of RNA binding or modification (termed ‘toeprint’ profiling) and includes an algorithm for handling multimapped reads using a Gibbs sampler approach to iteratively infer a single ‘best’ alignment for each read. While PROBer does include steps to handle multimapped reads, it was not developed specifically for TEs, and thus has not been tested on highly repetitive regions, such as TEs that are very close to the consensus.

## 8. DNA methylation-sequencing

We have detailed several methods to asses differential expression, and protein binding in the context of repetitive elements. However, a critical component to the understanding of transposon biology is the analysis of DNA methylation as it is the main mechanism by which transposons are transcriptionally silenced long term [113]. To assess DNA methylation, particularly the 5-methylcytosine (5-mC) modification, several techniques have been developed and compared [114]. In brief, the most common method to assess DNA methylation is bisulfite sequencing: whole-genome DNA sequencing following bisulfite conversion of all non-methylated cytosine residues to uracil. Bisulfite sequencing-based methods can be non-directional [115], or directional [116] allowing one to reduce the ambiguity of strand of origin. One of the first analysis pipelines developed for high-throughput bisulfite sequencing was in *Arabidopsis* [116] and analysis was performed in conjunction with sRNA-seq datasets. In this pipeline, ambiguously mapped reads were discarded by mapping to a repeat masked version of the genome, a technique once commonly used in animal systems to reduce mapping ambiguity in the context of bisulfite induced C > T conversions [117]. Bisulfite sequencing analysis differs significantly from other analysis pipelines in that often two reference genomes are used, one which contains converted cytosines in addition to the original reference genome. In this context, what are considered ambiguous reads are those reads which map to both the converted and unconverted reference genomes. This compounds the difficulty of assigning multimapped reads, such that many published bisulfite sequencing software packages choose not to include multimapped reads to avoid this confounded ambiguity (electronic supplementary material, table S1). The most commonly used pipelines for bisulfite sequencing reads including BSMAP [118], Bismark [119], MOABS [120] and BS-Seeker3 [121], none of which include probabilistic handling of multimapper reads. For a more comprehensive list of non-TE-specific methylation pipelines, please see the review by Adusumalli *et al*. [122] and the supplemental material of a recently published pipeline, Bicycle [123]. Here, confounding between ambiguity in bisulfite conversion rates, non-reference polymorphisms and read non-uniqueness can complicate the statistical tests used to determine if a site in the genome is differentially methylated. Thus, this represents an area of computational genomics that could benefit greatly from further development.

Because DNA methylation is a critical mechanism by which transposons are silenced, several groups have used new methods to improve methylation analysis for TEs. TEPID [124] and epiTEome [125] were designed to improve analysis of TE methylation levels by including the analysis of split reads that cross junctions between TEs and uniquely mappable genome regions. An approach employed to asses the low mappability of young TEs, like L1-Ta, in the human genome was repurposed to align bisulfite reads to a consensus sequence as described in Shukla *et al*. [126]. One interesting method to improve methylation analysis is to first rigorously determine the average bisulfite conversion rates genome-wide, then use this as a parameter to tease apart mapping ambiguities from differences in conversion rates, as done by Noshay *et al*. [127]. Despite these improvements, DNA methylation analysis is still a difficult bioinformatic challenge that would benefit from further study.

## 9. Single-cell RNA-sequencing

**Figure 3.**
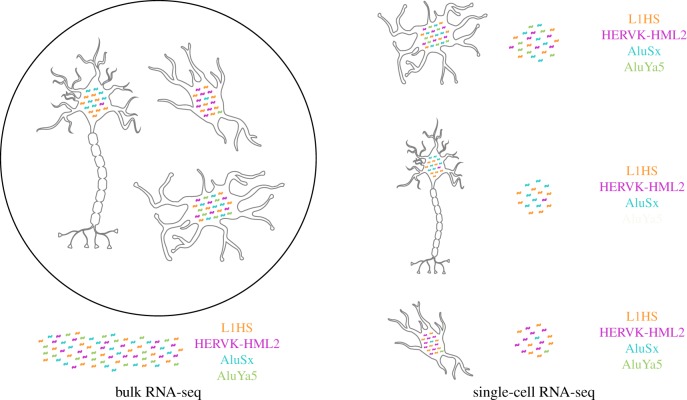
Comparison of bulk RNA-seq versus single-cell RNA-seq. Heterogeneity in expression profiles across cell types is masked by bulk sequencing methods. Transposable element (TE) expression may vary across cell types, between cells of the same type, and within the same cells across time. Single-cell methods are necessary to reveal this heterogeneity, but software for single-cell data analysis is not currently optimized for handling TEs.

All of the software described above has been geared towards genomics datasets generated from bulk tissue samples. However, bulk profiling of heterogeneous cell populations only provides averages that obscure underlying variability of TE expression across cell types, as illustrated in figure 3. This problem is further amplified when aggregating transcriptional signal across numerous loci within high copy-number TE families. It remains largely unknown how TE de-repression varies between individual cells, what factors drive such differences, and how this variability might affect cellular phenotypes. Single-cell RNA-sequencing (scRNA-seq) promises to answer some of those questions and has already redefined our knowledge about tissue composition and gene regulatory networks [128]. While its broad application has so far been largely restricted to the study of gene activity patterns, a few pioneering studies have used first-generation protocols to identify TE expression dynamics across single pre-implantation embryonic cells [129,130]. Those early efforts were largely limited by small cell numbers, high sequencing burden per cell, and lack of molecular barcode counts to estimate true transcriptional output, thus preventing broad-scale adaptation. Since then, the increasing demand in single-cell transcriptome data has seen an unprecedented expansion of available scRNA-seq protocols with considerably improved throughput, robustness, and error-rates [131]. One such publication was by Guo *et al*. [132], where the number of cells were scaled up allowing for investigation into TE dynamics in spermatogenesis.

**Figure 4.**
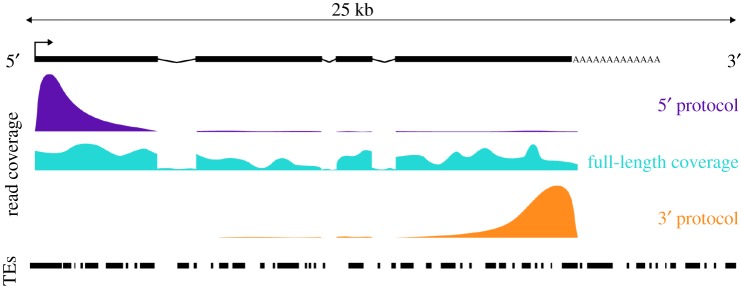
Impact of different RNA-seq library strategies on read coverage along a TE-derived transcript with exon/intron structure (top). TE intervals are shown at the bottom. Briefly, scRNA-seq protocols that offer full-length transcript coverage provide the best means to identify full length transcribed TEs in a locus-specific manner, but this method suffers from noise owing to intronic TEs in host genes that might be mistaken for expressed TE transcripts as well as the inability to barcode individual mRNA molecules. 5′- and 3′-based protocols allow for barcodes that enable mRNA molecule counting, with 5′ protocols also offering the ability to detect TE transcripts originating from proper TE promoters.

Despite such experimental advancements, inherent design principles of scRNA-seq protocols that cooperate with the well-known challenges of TE transcriptome analysis have so far prevented their common application for the study of TE expression at single-cell resolution (figure 4). For example, many popular methods quantify RNA molecules at the 3′ end of polyadenylated mRNAs [133–137] and therefore depend on accurate reference models to bridge the gap between polyadenylation sites and the corresponding transcript isoform and/or promoter. This is problematic for TE-derived transcripts, which are generally poorly annotated in many species. While protocols with full-length transcript coverage might alleviate some of those problems, the naive assignment of reads to the nearest TE interval can still lead to erroneous assignment, misattribution of intronic reads from unprocessed pre-mRNAs, and hence misinterpretation of TE de-repression. Full-length protocols additionally suffer from higher sequencing burden, often lack of unique molecular identifiers to account for PCR duplicates, and potentially higher background TE read coverage owing to intronic signal originating from pre-mRNAs [138,139].

A potential solution to minimize misattribution problems are 5′ end based scRNA-seq protocols that incorporate a template switch oligo (TSO) towards the start of transcription initiation [140,141]. Although incomplete processing and premature TSO incorporation during library preparation might vary between transcripts and cells, such protocols have already been successfully used to map alternative transcription start sites between individual cells [142]. Importantly, a recent study also demonstrated its utility to quantify unexpected variability in TE promoter activity between thousands of single cancer cells following epigenetic therapy [143]. However, the problem of premature TSO incorporation, combined with the pervasive nature of TEs, and technical noise inherent to all current scRNA-seq protocols requires dedicated strategies to mitigate the danger of spurious estimates of TE cell-to-cell variation. To the best of our knowledge, no peer-reviewed computational pipeline currently combines such features with the reliable quantification of TEs at single-cell resolution, but unpublished efforts already aim to facilitate TE single-cell analysis for a wide array of available scRNA-seq protocols (https://tanaylab.github.io/Repsc/). With the continuous methodological advancements and the increasing interest in TE biology, we anticipate a rapid progress towards the routine quantification of TEs in individual cells that will be accompanied by the discovery of unprecedented heterogeneity in TE transcription patterns.

## 10. Conclusion

### (a) What is now doable?

The last years have seen a general improvement in sequencing read length, making it possible to study the majority of TEs in a genome-wide fashion. For particularly young and less diverged families, we have discussed at length the strides made in genome biology to address the difficulties of treating ambiguously mapped sequencing fragments for differential expression and binding analyses. In the context of highly repetitive regions of the genome, these difficulties are compounded, particularly for the most active TEs, which remain close to their consensus sequence and thus are the most difficult to map. The greatest progress has been made with RNA-seq data analysis, as we have progressed from using simple fractional assignments of multimapped reads within genes to approaching true locus-specific resolution in the most repetitive regions of the genome—such as the L1HS subfamily, active Alu families and composite SVA elements. Progress has been made in the realm of sRNA analysis as these improved algorithms for RNA-seq analysis have now been incorporated into sRNA-seq data analysis pipelines. In immunoprecipitation based assays, for ChIP- and CLIP-seq datasets, efforts have been made to use probabilistic read redistribution for peaks within repetitive regions, but challenges remain.

### (b) What is still hard?

sRNA-seq data contains a large proportion of multimapped reads, and while significant effort has been put forth to leverage advanced iterative statistical methods for novel sRNA discovery and target prediction, these methods have not been as widely applied to sRNA-seq transcript quantification. This may be attributed to the tight distribution of sRNA reads across their mapping loci, making it difficult to garner locus-specific information from adjacent reads. Moreover, these much shorter reads (18–30 nt) are intrinsically less unique in the genome than longer sequences.

In ChIP-seq data, the expected profile of read distributions can vary widely from the typically tall, narrow peaks associated with most transcription factor profiles or RNA-binding proteins to the broader, shorter and noisier peaks associated with some marked histones, such as H3K9me3. Algorithms have been developed to address both types of ChIP-seq profiles. Yet the lines between these categories can be blurred, and there is a large trade-off between the window size in peak calling and the ability to use uniquely mapped reads to probabilistically reassign all other reads to a particular locus. One area of active research for broader regions would be to incorporate multimapped reads into segmentation models which allow for the detection of changes in peak landscape, as opposed to simply calling the absence or presence of individual peaks.

scRNA-seq represents one of the newest genomic assays to be used for TE expression profiling, and as such, remains an area of greatest need for improvements in software packages specifically designed to handle the complexities inherent in TE genomics. Efforts are already underway, but as yet no published software packages for scRNA-seq are available. That said, many standard scRNA-seq packages could be adapted for this use, as in the example protocol described above. However, as discussed in detail, differences in the experimental protocols used to generate scRNA-seq libraries will have a large impact upon the interpretability of the data, and this is particularly problematic for TE expression analysis.

Two types of analysis which largely do not include multimapped reads are assays for transposase accessible chromatin using sequencing (ATAC-seq) [144] and Hi-C [145], an extension of chromosome conformation capture (3C). The read distributions for ATAC-seq data greatly resemble those of ChIP-seq and this analysis encounters similar computational difficulties when studying repetitive regions of the genome. Fortunately, as this analysis is similar to ChIP-seq there has already been significant effort which could be incorporated into ATAC-seq analysis. Adapting Hi-C pipelines to take into account multimapped reads is still a difficult task as this type of analysis already requires the resolution of chimeric reads representing genomic proximity. mHiC [146] has been developed to address this issue, but the relative sensitivity to highly repetitive transposon regions is unclear. Significant work has been done using these methods to address the role of transposons in genome architecture and the transition from the embryonic cell state to early embryonic-like cells [99,147,148]. These analyses can only improve as better methods for handling repetitive reads are included.

### (c) What new technology needs to be developed?

Long-read sequencing technologies promise to solve many issues inherent in the assays described above. Once the issues with throughput and error rates can be solved, long-read sequencing would enable the isolation of entire transcripts and, if correctly barcoded, would also allow for accurately calibrated expression estimates. These technologies could also be combined with antibody-based pulldowns and endonuclease-based footprinting assays, to accurately call *cis*-regulatory regions derived from TEs. Finally, long-read genome resequencing assays that sequence through highly repetitive genome regions may allow for better genomic annotations that will benefit all of the applications described above. To this end, not only must new experimental protocols be developed which emphasize longer reads but new computational pipelines must also be developed to ensure that these long read analysis pipelines properly handle and account for the complications inherent in addressing TE genomics.

## Supplementary Material

Table S1: Software packages discussed in this review

## Data accessibility

This article has no additional data.
